# Development of a novel risk score to predict mortality in patients admitted to hospital with COVID-19

**DOI:** 10.1038/s41598-020-78505-w

**Published:** 2020-12-07

**Authors:** Ying X. Gue, Maria Tennyson, Jovia Gao, Shuhui Ren, Rahim Kanji, Diana A. Gorog

**Affiliations:** 1 University of Hertfordshire, College Lane, Hatfield, AL10 9 AB UK; 2 East and North Hertfordshire NHS Trust, Lister Hospital, Stevenage, Hertfordshire SG1 4AB UK; 3 National Heart and Lung Institute, Imperial College, Dovehouse Street, London, SW3 6LY UK

**Keywords:** Diseases, Infectious diseases, Viral infection

## Abstract

Patients hospitalised with COVID-19 have a high mortality. Identification of patients at increased risk of adverse outcome would be important, to allow closer observation and earlier medical intervention for those at risk, and to objectively guide prognosis for friends and family of affected individuals. We conducted a single-centre retrospective cohort study in all-comers with COVID-19 admitted to a large general hospital in the United Kingdom. Clinical characteristics and features on admission, including observations, haematological and biochemical characteristics, were used to develop a score to predict 30-day mortality, using multivariable logistic regression. We identified 316 patients, of whom 46% died within 30-days. We developed a mortality score incorporating age, sex, platelet count, international normalised ratio, and observations on admission including the Glasgow Coma Scale, respiratory rate and blood pressure. The score was highly predictive of 30-day mortality with an area under the receiver operating curve of 0.7933 (95% CI 0.745–0.841). The optimal cut-point was a score ≥ 4, which had a specificity of 78.36% and a sensitivity of 67.59%. Patients with a score ≥ 4 had an odds ratio of 7.6 for 30-day mortality compared to those with a score < 4 (95% CI 4.56–12.49, p < 0.001). This simple, easy-to-use risk score calculator for patients admitted to hospital with COVID-19 is a strong predictor of 30-day mortality. Whilst requiring further external validation, it has the potential to guide prognosis for family and friends, and to identify patients at increased risk, who may require closer observation and more intensive early intervention.

## Introduction

The Corona virus disease 2019 (COVID-19) pandemic, originating in China in late 2019, has affected over 6.8 million people with more than 400,000 deaths worldwide^1^. This disease, caused by the severe acute respiratory syndrome coronavirus 2 (SARS-CoV-2)^2^, has impacted and placed tremendous strains even on the best healthcare systems in the world, due to its high infectivity and transmission rates and in some patients, a rapid clinical deterioration resulting in acute respiratory distress syndrome and thrombotic complications, with high in-hospital morbidity and mortality. A meta-analysis involving 4659 hospitalised patients with an average age of 59.8 years showed a mortality rate of 26%, with advancing age associated with increasing risk^3^. A recent survey of 20,133 hospitalised patients with COVID-19 in the UK with a mean age of 79 years showed that only 41% of patients were discharged alive, 26% died, and 34% were still in hospital^4^. There is, therefore, an urgent need to identify patients admitted to hospital with COVID-19 who are at increased risk of death, so that these patients can be observed more closely, receive earlier and more aggressive medical intervention, and to guide prognosis and possibly also resource allocation.

Since the recognition of the potential life-threatening nature of this disease, clinicians and researchers have come together all over the world to collate data and identify patients at risk of a more severe clinical course of COVID-19. Several observational studies have identified demographic and clinical risk factors such as age, male gender, hypertension and obesity as risk factors for severe disease progression and death^3^. Earlier reports also indicate that patients with comorbidities including chronic obstructive pulmonary disease, cardiovascular disease, hypertension, and patients presenting with dyspnoea are vulnerable to more severe morbidity and mortality after infection^5,6^. Most such risk factors have been identified from detecting people in the general population, not in hospital, who are at increased risk of becoming infected with COVID-19 or being admitted to hospital with the disease. These risk factors therefore identify those patients in the community who are most vulnerable, but not mortality risk in-hospitalised patients with COVID-19. Since the pandemic, several risk scores including those specific to COVID-19 such as the Brescia-COVID^7^ and more generic sepsis-related risk scores like the SOFA (Sequential Organ Failure Assessment)^8^ or SIC (Sepsis induced coagulopathy)^9^ score have been used in clinical situations to guide management of COVID-19 patients. Furthermore, whilst a number of haematological, biochemical and radiological characteristics have also been associated with adverse prognosis^3,10^, there is currently no easy-to-use prognostic risk score to calculate 30-day mortality in patients admitted to hospital with COVID-19.

It was our aim to develop a clinically friendly, easy-to-use prognostic score to predict 30-day mortality in patients admitted to hospital with COVID-19.

## Methods

We conducted a single-centre retrospective cohort analysis of the electronic records of all patients admitted to East and North Hertfordshire NHS Trust, a large district general hospital in the United Kingdom, over a period of 3 months (10th March 2020 to 30th May 2020) with symptoms of COVID-19, as part of an approved service evaluation by the Research and Development board of the East and North Hertfordshire NHS Trust. Since this was a service evaluation by clinicians with genuine clinical access to patient records, and since only anonymised data was analysed, the Research and Development board of the East and North Hertfordshire NHS Trust waived the requirement to see patient consent. All methods were carried out in accordance with relevant institutional and Good Clinical Practice guidelines and regulations.

### Patient population

Clinical data of all consecutive patients who were admitted to the hospital and tested positive for SARS-CoV2 on nasal or oropharyngeal swabs were retrospectively collected. Only patients who were admitted with clinical symptoms of COVID-19 were included. We excluded patients who were either admitted with non-COVID-19 symptoms and had incidental asymptomatic diagnosis (such as those for example with hip fracture without clinical features of COVID-19) and also excluded those with COVID-19 who were discharged from either ambulatory care or the emergency room, without admission. We excluded patients who had a terminal diagnosis due to a pre-existing condition prior to admission.

### Data collection

From an available registry of all patients testing positive for SARS-CoV2 in our institution, we identified those who were admitted, and then four clinicians screened this residual cohort to identify those that met the inclusion criteria. Any queries were resolved by consensus. Electronic patient records were accessed to extract basic demographics (age, gender, ethnicity), medical history, admission observations, admission blood test results including full blood count, biochemical profile, high-sensitivity C-reactive protein (hs-CRP), coagulation screen and outcome data.

### Calculation of qSOFA and mSIC scores

For each patient, the Quick Sequential Organ Failure Assessment (qSOFA) score^11,12^ was calculated from data on admission. This score, calculated from a combination of binary variables derived from clinical observations (Glasgow Coma Scale < 15, respiratory rate > 22 and systolic blood pressure < 100), was calculated from clinical assessment recorded on admission (Table 4).

In patients with sepsis, coagulopathy is a known predictor of adverse outcome. The Sepsis-Induced Coagulopathy (SIC) scoring system was the first scoring system specifically designed for coagulation disturbances in sepsis. It is easy to calculate and has a high predictive value for 28-day mortality^9,13,14^. The score incorporates points for platelet count, international normalized ratio (INR) and SOFA score. However, the SOFA score, in contrast to the qSOFA score, is designed for patients with severe sepsis admitted to the intensive care unit (ICU), and requires the measurement of the partial pressure of arterial oxygen. This is not applicable to patients who are not *in extremis* and not requiring ICU admission. Thus, in our cohort of patients, to calculate the SIC score, instead of using the SOFA score, we modified the SIC score to incorporate instead the qSOFA score, as above, and we refer to this as the modified Sepsis-Induced Coagulopathy (mSIC) score (Table 4). The mSIC score was obtained using the admission INR (score of 0 for INR < 1.2, 1 for INR between 1.2 and 1.4 and 2 for INR > 1.4) and platelet count (score of 0 for platelets > 150 × 10^9^/L, 1 for platelets between 100 to 150 × 10^9^/L and 2 for < 100 × 10^9^/L) in conjunction with the qSOFA score.

### Outcome

The primary outcome of interest was 30-day mortality. This data was captured from electronic medical records.

### Statistical analysis

Categorical variables were summarised as proportion (number, percentage) and continuous variables as median (interquartile range, IQR). Binary variables were compared using Fisher’s exact test whilst continuous variables were compared using Wilcoxon rank sum test. Variables which were significantly different between patients that survived and those that died were entered into a univariate logistic regression analysis. Odds ratios (ORs) with 95% confidence interval (CI) and p-values were obtained. Continuous variables which were found to be significantly related to mortality were converted to a binary value, with the cut-point determined by receiver operating curve (ROC) analysis to obtain the area under the curve (AUC, c-statistic) and to identify the highest predictive value. Variables which remained statistically significant were entered into a multivariable logistic regression model to identify variables which remained independently associated with mortality. Using the ORs obtained in the multivariable regression model, we produced a COVID-19 mortality prediction score. The predictive ability of the COVID-19 mortality score for the primary outcome was assessed with ROC analysis and the c-statistic reported. Significance was taken as < 0.05. Statistical analyses were performed using Stata 15 software (StataCorp, College Station, Texas, USA).

## Results

A total of 486 in-patients tested positive for COVID-19 during this period, of whom 316 met the inclusion criteria. Out of these patients, 145 (46%) died within 30 days of hospital admission. Non-survivors were significantly older (81 [74–88] vs. 67 [54–80] years), were more likely to be male (72.2% vs. 54.7%), with a history of hypertension (60% vs. 44.4%), coronary artery disease (22.1% vs. 9.4%) and atrial fibrillation (22.8% vs. 12.9%). They were more likely to be on oral anticoagulants on admission (24.8% vs. 10.5%). The admission blood profile showed significantly lower haemoglobin and higher white cell count, neutrophils, hs-CRP and INR in those who died compared to those who survived (Table 1). The qSOFA and mSIC score were significantly lower in those who survived, compared to those who died.

**Table 1:** Baseline patient characteristics on admission.

	Survivors (n = 171)	Non-survivors (n = 145)	p-value
Age	67 (54–80)	81 (74–88)	**< 0.0001**
Male sex	84 (54.7)	104 (72.2)	**0.001**
**Ethnicity**			
Caucasian	155 (90.6)	134 (92.4)	0.687
Asian	10 (5.9)	4 (2.8)	0.273
Black	4 (2.3)	7 (4.8)	0.356
Mixed	2 (1.2)	0 (0)	0.50
Hypertension	76 (44.4)	87 (60.0)	**0.007**
Diabetes	50 (29.2)	42 (29.0)	1.00
Coronary artery disease	16 (9.4)	32 (22.1)	**0.003**
Dyslipidaemia	45 (26.3)	45 (31.0)	0.383
Heart failure	19 (11.1)	30 (20.7)	**0.028**
AF	22 (12.9)	33 (22.8)	**0.025**
CKD	18 (10.5)	20 (13.8)	0.391
Oral anticoagulants	18 (10.5)	36 (24.8)	**0.0008**
**Admission bloods**			
Hb, g/L	133 (118–146)	126 (110–140)	**0.0152**
WCC, × 10^9^/L	7.3 (5.2–10.4)	8.9 (5.8–12.1)	**0.0032**
Neutrophil, × 10^9^/L	5.2 (3.82–8.6)	7.21 (4.46–10.46)	**0.0024**
Lymphocytes, × 10^9^/L	0.86 (0.62–1.2)	0.83 (0.59–1.32)	0.8914
Platelets, × 10^9^/L	210.5 (169–277)	206 (153–292)	0.2948
Hs-CRP, mg/L	72 (41–153)	121 (64–210)	**0.0004**
INR	1 (1–1.1)	1.1 (1–1.2)	**0.0001**
**Severity scores**			
qSOFA score	1 (0–1)	1 (1–2)	**< 0.0001**
mSIC score	1 (0–1)	2 (1–3)	**< 0.0001**
COVID-19 mortality score	2 (1–3)	4 (3–5)	**< 0.0001**

*AF* Atrial Fibrillation, *CKD* Chronic Kidney Disease (defined as eGFR of less than 60 mL/min/1.73 m^2^), *Hb* Haemoglobin, *WCC* White cell count, *CRP* C-reactive protein, *INR* International normalised ratio, *qSOFA* quick Sepsis-related Organ Failure Assessment score, *mSIC* modified Sepsis-Induced Coagulopathy score.

Bold values indicate p < 0.05.

### Predictors of 30-day mortality

Univariate logistic regression was performed for those clinical and pathological variables that differed significantly on admission between patients who died and those who survived (Table 2), with subsequent ROC analysis for continuous variables to identify the optimal cut-point for regression analysis. Age had an AUC of 0.723 with the optimal cut-point of 75 years. Haemoglobin (AUC 0.421), white cell count (AUC 0.596), neutrophil count (AUC 0.599) and hs-CRP (AUC 0.616) had poor predictive ability as demonstrated on AUC analysis and were therefore not selected for logistic regression analysis.

**Table 2:** Univariate logistic regression analysis of variables that were significantly different between survivors and non-survivors.

	Odds ratio	95% CI	p-value
Age ≥ 75 years	4.38	2.73 – 7.06	< 0.001
Male	2.20	1.37 – 3.53	0.001
Hypertension	1.88	1.20 – 2.94	0.006
Coronary artery disease	2.74	1.44 – 5.24	0.002
Heart failure	2.09	1.12 – 3.89	0.021
AF	2.00	1.10 – 3.61	0.022
Oral anticoagulants	2.81	1.51 – 5.20	0.001
mSIC score	2.35	1.81 – 3.04	< 0.001

*AF* Atrial Fibrillation, *mSIC* modified Sepsis-Induced Coagulopathy score.

As the mSIC score incorporated the qSOFA score and INR, these variables were not included in the regression model even though they were significantly different when comparing survivors and non-survivors. These were then entered into a multivariable logistic regression model, which showed that the variables of age ≥ 75, male sex and mSIC score remained independently predictive of 30-day mortality (Table 3).

**Table 3:** Multivariable logistic regression analysis.

	Odds ratio	95% CI	p-value
Age ≥ 75 years	3.84	2.25–6.83	**< 0.001**
Male	1.88	1.06–3.21	**0.026**
Hypertension	1.40	0.82–2.40	0.218
Coronary artery disease	1.62	0.76–4.07	0.273
Heart failure	0.87	0.39–2.13	0.750
AF	0.49	0.35–1.58	0.137
Oral anticoagulants	2.08	0.81–5.41	**0.130**
mSIC score	2.37	1.79–3.14	**< 0.001**

*AF* Atrial Fibrillation, *mSIC* modified Sepsis-Induced Coagulopathy score.

Bold values indicate p < 0.05.

### Development of a COVID-19 mortality risk score

From those variables that remained independently predictive of 30-day mortality on multivariable regression analysis, we developed a COVID-19 mortality score by weighting each characteristic based on the OR. Based on this, age ≥ 75 was given a weighting of 2 points and male sex a weighting of 1 point, with the mSIC score allocated points as shown in Table 4, to give a final score out of 10.

**Table 4:** The COVID-19 mortality score for predicting 30-day outcome in hospitalised patients.

COVID19 mortality score			
Components			Points
**Modified sepsis-induced coagulopathy (mSIC) score**	INR	≤ 1.2	0
		> 1.2 and ≤ 1.4	1
		> 1.4	2
	Platelet count	≥ 150	0
		≥ 100 and < 150	1
		< 100	2
	qSOFA score	GCS < 15	1
		RR > 22	1
		Systolic BP < 100	1
**Age**	≥ 75 years old		2
**Gender**	Male		1

The score is derived from a multivariable regression analysis of independently predictive variables.

**Figure 1:**
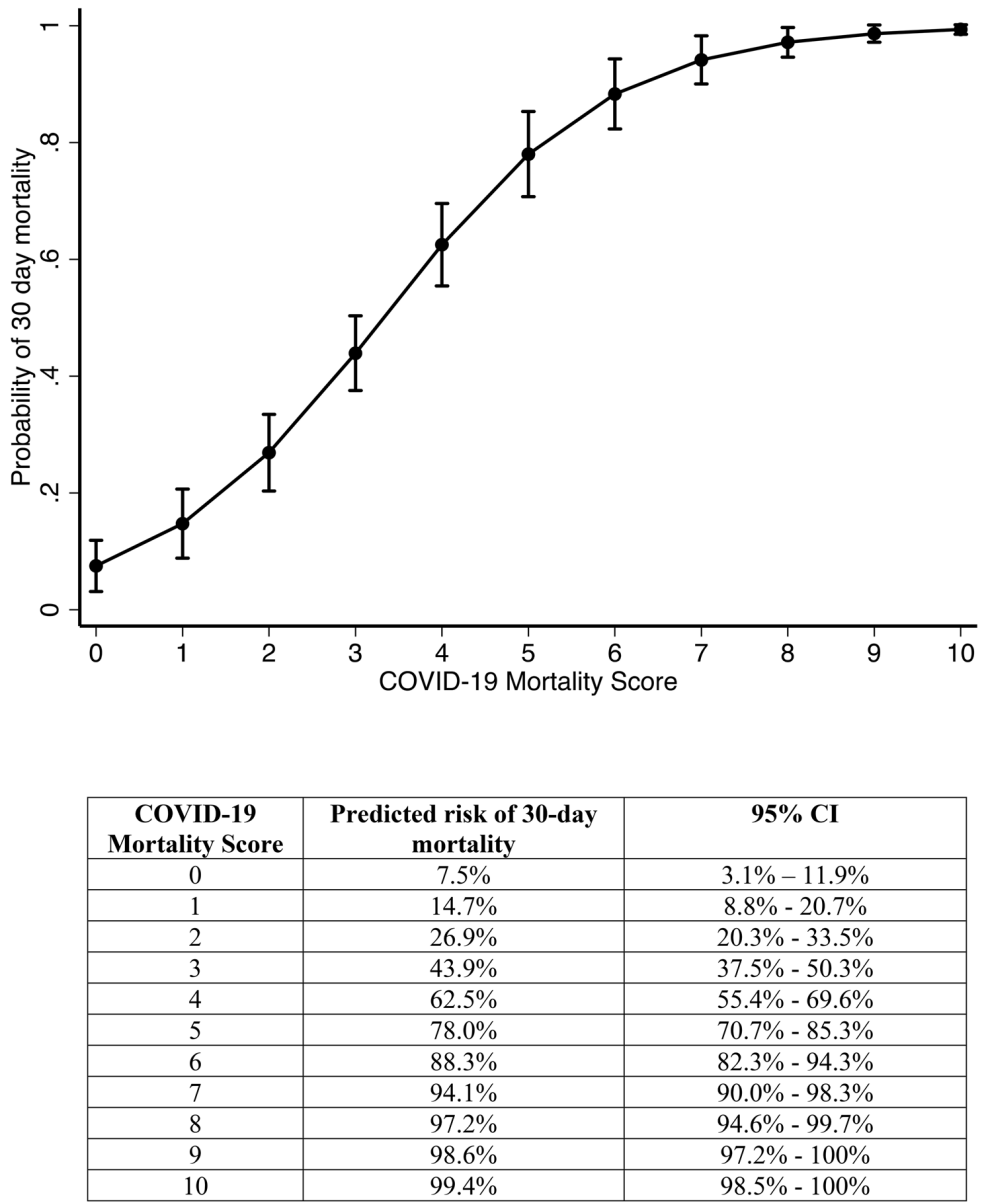
The COVID-19 mortality score adjusted prediction of 30-day mortality, with 95% CI.

As the COVID-19 score increased, the 30-day mortality increased (Fig. 1). The score had an AUC of 0.793 (95% CI 0.745–0.841) for predicting mortality, which is an improvement on both the mSIC (AUC 0.717, 95% CI 0.664–0.770) and the qSOFA (AUC 0.691, 95% CI 0.638–0.743) scores. The optimal cut-point with the highest combined sensitivity and specificity was a score of ≥ 4, which has a specificity of 78.36% and a sensitivity of 67.59%, with a positive predictive value of 72.6% and negative predictive value of 74%. This means that patients with a score of ≥ 4 have a 73% chance that they will die within 30 days, and those with a score < 4 have a 74% chance of still being alive at 30 days (Table 5). A score of ≥ 4 had an OR of 7.55 (95% CI 4.565–12.493, p < 0.001) when compared to scores of < 4.

**Table 5:** Usefulness of the COVID-19 mortality score for predicting 30-day death.

COVID-19 mortality score	Sensitivity (%)	Specificity (%)	PPV (%)	NPV (%)
≥ 1	100	12.3	50.8	100
≥ 2	95.2	35.1	55.6	89.6
≥ 3	80	60.2	63.0	78.0
≥ 4	67.6	78.4	72.6	74.0
≥ 5	39.3	93.6	83.8	64.5
≥ 6	13.1	97.7	82.6	57.0
≥ 7	3.5	100	100	56.8
≥ 8	1.4	100	100	54.5

*PPV* positive predictive value, *NPV* negative predictive value.

## Discussion

The ability to determine the risk of adverse outcome for people infected with SARS-CoV-2 admitted to hospital is very important both for the individual, in terms of highlighting the need for more intensive observation and earlier medical intervention, as well as to aid health care professionals to guide patients’ families in terms of providing some objective, although guarded, assessment of prognosis.

The COVID-19 mortality risk score we have developed provides a simple and objective way to assess 30-day mortality risk in patients admitted to hospital. The score is a good predictor of adverse outcome, with an AUC of 0.793 (95% CI 0.745–0.841) and 30-day mortality increases as the COVID-19 score increases (Table 5). Patients with a score of ≥ 4 had an almost eightfold 30-day mortality risk when compared to those with a score of < 4 (OR 7.55; 95% CI 4.56–12.49, p < 0.001). Physicians find positive and negative predictive values good ways to communicate risk, and those with a score of ≥ 4 have a 73% chance that they will die within 30 days, and those with a score < 4 have a 74% chance of still being alive at 30 days.

Important strengths of our model are the inclusion of all-comers, the use of a hard clinical end-point, the ease-of-use and use of multivariable regression to select independent predictive markers. Such prognostic information can be very useful for both healthcare professionals and relatives, can be easily and quickly calculated, and easily understood by lay individuals. The score is very simple to calculate, does not require a calculator or a normogram, and all the variables used in the score are routinely available, with no requirement for specific data from specialised tests.

Most risk score predictive models developed for COVID-19 to date have been designed for assessing risk in the community and risk of admission to hospital^15^. One of the largest cohorts (35,463 patients) used to develop an in-hospital mortality score in COVID-19 is the 4C mortality score^16^. It utilises several variables including patient characteristics (age, gender and comorbidities), admission status (respiratory rate, oxygen saturation and Glasgow Coma Scale score) and blood parameters (urea and CRP). The calculation is easy with a good AUC (0.79 in development cohort and 0.77 in the validation cohort). This is similar to our risk score with the difference being that it predicts in-hospital, rather than 30-day, mortality. Some small studies have aimed to predict severity of disease progression in hospital (the latter was not clearly defined, and not mortality-based)^15,17,18^. A report in 208 patients from China, with a median age of only 44 years, described a novel scoring system to predict disease progression based on underlying comorbidity, age, higher LDH and lower lymphocyte count^19^. However, disease progression was variably defined as one or more of respiratory rate ≥ 30 breaths/min, resting oxygen saturation ≤ 93%, PaO_2_/FiO_2_ ≤ 300 mmHg, mechanical ventilation or worsening of lung CT findings. Mortality was not reported, but likely to be low given the age-group. The COVID-GRAM score^20^ was developed from a cohort of 1590 patients in China to predict progression to critical illness which included intensive care admission, invasive ventilation or death. Although the score has shown an AUC of 0.88 in predicting risk of critical illness, the complexity including the requirements for a web-based calculator and various parameters including lactate dehydrogenase levels (which are not routinely measured in all hospitals) makes the score harder to use. Furthermore, the definition of “critical illness” can be nebulous and is not a hard clinical endpoint. Another early mortality risk score for hospitalised patients was developed from only 75 patients with COVID-19 and based on only age and C-reactive protein level^21^. On the other hand, our risk score uses a hard clinical end-point and has assessed a larger generalised cohort with more comorbidities, which we feel more closely resembles the typical cohort admitted to hospital with COVID-19.

Hu et al*.* explored the use of alternative scores such as the Modified Early Warning Score and Rapid Emergency Medicine Score in critically ill COVID-19 patients, which yielded satisfactory AUC (0.677 and 0.833 respectively)^22^. This scoring system is applicable to only critically-ill hospitalised patients, whereas our score predicts mortality in all hospitalised patients. Furthermore, the reported mortality by Hu et al*.* was much lower than in our cohort (18% vs 46%), which is surprisingly low, given reported mortality rates in other critically ill COVID-19 cohorts, suggesting a rather loose definition of “critical illness”.

Our cohort of patients had a higher 30-day mortality rate compared to a similar study by Giacomelli et al*.* (46% vs. 20.6%)^23^. This could be explained by the older age of our cohort (median 75 vs. 61 years) and the different setting- ours being a large district general hospital whilst the other was a specialist infectious disease hospital. However, our mortality rate is reflective of data obtained in 16,749 hospitalised UK patients of similar age to ours^24^. Baseline characteristics predictive of mortality in our cohort were similar to those previously reported, namely older age, male gender, hypertension and cardiovascular disease. As many of these conditions co-exist in elderly patients, the multivariable logistic regression used provides evidence that after adjustment, the only variables that remain independently predictive of 30-day mortality were age, gender and the mSIC score. Individuals with diabetes and black, Asian and minority ethnic (BAME) groups were under-represented in our cohort and so suitable conclusions on these risk factors cannot be drawn from our data.

Importantly, no risk score calculator will be 100% accurate. Therefore, such a scoring system can at best serve as an adjunct to decision-making during admission and can be used to identify those at high risk who may require more careful review and earlier intervention, and to guide and explain prognosis to relatives who may find that being quoted an objective survival rate based on the score may help better prepare them for the future.

### Limitations

There are a number of limitations in our study. Firstly, as retrospective study, bias and confounders that are not identified cannot be addressed, and patients at the highest risk may be deemed too sick for maximal intervention and may be denied ICU treatment. Prediction of mortality was made retrospectively with knowledge of outcome data which introduces bias. The predictors and their assigned weights in the final model did not exactly correspond to those reported in the multivariable analysis, but have been rounded to whole numbers to make the score easier to use. Ours is a relatively small cohort from a single UK centre, and we cannot know whether our predictive model applies equally to other geographies and healthcare models, to patients with diabetes, or to those in BAME groups or other ethnicities. Finally, our proposed predictive model requires external, independent validation in a large prospective cohort.

## Conclusion

We have developed a simple, easy-to-use risk score calculator for patients admitted to hospital with COVID-19, which is a strong predictor of 30-day mortality. Whilst the score requires further external validation, it has the potential to guide prognosis for family and friends and to identify patients at increased risk, who may require closer observation and more intensive or earlier intervention.

## Abbreviations

AUC: Area under the receiver operating curve
CI: Confidence interval
COVID-19: Corona virus disease 2019
ICU: Intensive care unit
IQR: Interquartile range
mSIC: Modified sepsis-induced coagulopathy
OR: Odds ratio
PPV: Positive predictive value
qSOFA: Quick sequential organ failure assessment
ROC: Receiver operator characteristic curve
SARS-CoV-2: Severe acute respiratory syndrome coronavirus 2
SIC: Sepsis-induced coagulopathy
SOFA: Sequential organ failure assessment
